# Distinct Phenotypes of *Shank2* Mouse Models Reflect Neuropsychiatric Spectrum Disorders of Human Patients With *SHANK2* Variants

**DOI:** 10.3389/fnmol.2018.00240

**Published:** 2018-07-19

**Authors:** Ahmed Eltokhi, Gudrun Rappold, Rolf Sprengel

**Affiliations:** ^1^Max Planck Research Group “Molecular Neurobiology”, Max Planck Institute for Medical Research, Heidelberg, Germany; ^2^Department of Human Molecular Genetics, Institute of Human Genetics, Heidelberg University, Heidelberg, Germany; ^3^Institute for Anatomy and Cell Biology, Heidelberg University, Heidelberg, Germany

**Keywords:** SHANK2 domains, SHANK2 isoforms, *SHANK2* gene variants, autism spectrum disorder, intellectual disability, schizophrenia, *Shank2* knockout mice, behavioral tests

## Abstract

The SHANK scaffolding proteins are important organizers for signaling proteins in the postsynapse of excitatory neurons. The functional significance of SHANK proteins becomes apparent by the wide spectrum of neurodevelopmental and neuropsychiatric disorders associated with *SHANK* variants in human patients. A similar diversity of neuropsychiatric-like phenotypes is described for numerous *Shank2* and *Shank3* knockout (KO) mouse lines. In this review, we will focus on and discuss the experimental results obtained from different, but genetically related and therefore comparable, *Shank2* mouse models. First, we will describe the distinct *SHANK2* variant-mediated neurodevelopmental and neuropsychiatric disorders in human patients. Then we will discuss the current knowledge of the expressed SHANK2 isoforms in the mouse, and we will describe the genetic strategies used for generating three conventional and seven conditional *Shank2* mouse lines. The distinct impairments i.e., autistic-like and mania-like behavior and the alterations on the molecular, electrophysiological and behavioral levels will be compared between the different *Shank2* mouse models. We will present our view as to why in these mouse models a spectrum of phenotypes can arise from similar *Shank2* gene manipulations and how *Shank2* mutant mice can be used and should be analyzed on the behavioral level in future research.

## Introduction

Gene variants of the multi-domain postsynaptic scaffolding proteins included in the SHANK family (also known as ProSAP) are significantly associated with autism spectrum disorders (ASD). After the first publication of a *SHANK3* variant in human patients with ASD (Durand et al., 2007), numerous studies confirmed the close link between *SHANK* variants and ASD and intellectual disability (ID; see https://gene.sfari.org). In humans, three different genes, *SHANK* *1*, *2* and *3* produce several SHANK isoforms via internal promoters and alternative splice products (Lim et al., 1999; for review see Sala et al., 2015). SHANK proteins are master scaffolding proteins in the postsynapse of excitatory neurons. There, they are critically involved in the morphogenesis of spines (Sala et al., 2001). Within the spine, SHANK proteins form a net-like matrix structure (Baron et al., 2006; Hayashi et al., 2009) that serves as a scaffold for the organization of other postsynaptic proteins including N-methyl-D-aspartate receptors (NMDAR), L-α-amino-3-hydroxy-5-methyl-4-isoxazolepropionic acid receptors (AMPAR) and the metabotropic glutamate receptors (mGluRs; Sheng and Kim, 2011; Jacob and Weinberg, 2015; for review see Verpelli et al., 2012). The longest isoforms of SHANK1–3 contain several protein-protein interaction sites: the N-terminal ankyrin repeat-containing (ANK) domain, the Src homology 3 (SH3) domain, the PSD-95/Discs large/zona occludens (PDZ) domain, the proline-rich region (PRR) and the sterile alpha motif (SAM) in the C-terminal domain. The N-terminal ANK domain interacts with the cytoskeletal protein alpha-fodrin (Böckers et al., 2001) and the SH3 domain binds to glutamate receptor-interacting protein (GRIP), which is important for AMPAR trafficking (Lu and Ziff, 2005). The PDZ site in SHANKs can bind to the adaptor protein guanylate kinase-associated protein (GKAP; Naisbitt et al., 1999). GKAP itself can bind to the postsynaptic density molecule 95 (PSD95; Naisbitt et al., 1997) that is in contact with the glutamate gated ion channels, NMDAR (Kornau et al., 1995) and AMPAR (Kim et al., 2001; Uemura et al., 2004; for review see Boeckers et al., 2002). Most clearly the interaction of SHANK and Homer proteins via the PRR was noted (Naisbitt et al., 1999; Tu et al., 1999). This interaction allows the coupling of mGluRs to Ca^2+^ release from endoplasmic reticulum (ER) in the postsynaptic density. Cortactin, a molecule involved in actin polymerization, was also shown to bind to PRR (Ammer and Weed, 2008). The C-terminal SAM domain serves as a Zn^2+^ dependent dimerization domain for SHANKs (Baron et al., 2006; Gundelfinger et al., 2006).

**Figure 1:**
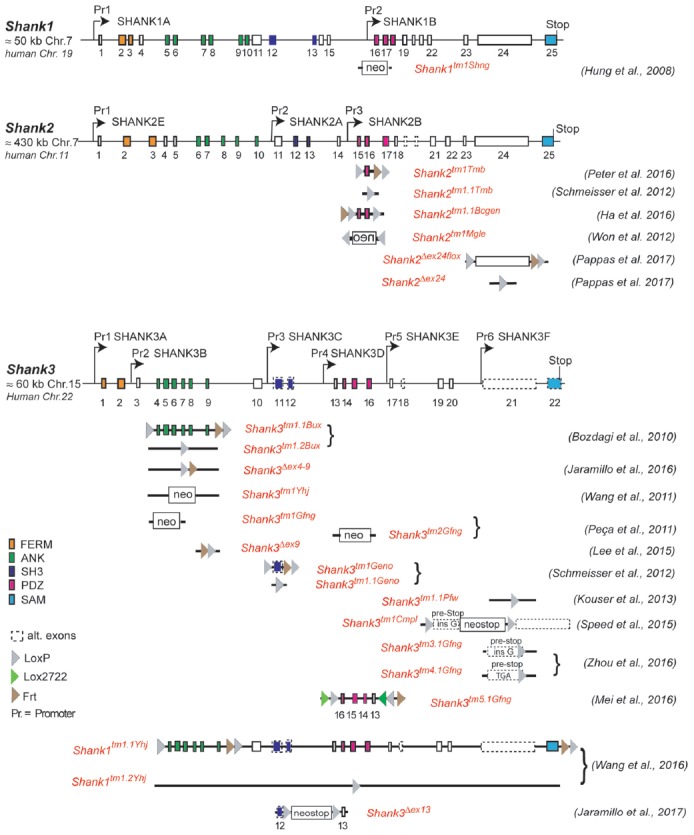
Gene structure and gene segment deletions in gene-targeted *Shank* mouse models. Structures of the mouse *Shank1*, *Shank2* and *Shank3* genes are depicted. Exons are given in rectangles; alternative spliced exons are in dashed lines. Positions of promoters (Pr) for the expression of the different isoforms of the *Shank1–3* gene loci are indicated. Positions of the neomycin (neo) selection marker, loxP, lox2722 and Frt sites in the targeted alleles are indicated. Targeted gene segments flanked by two loxP or two lox2722 elements can be removed or inverted by tissue-specific expression of Cre to generate a conditional *Shank* knockout (KO) or *Shank* knockin mouse models. neo = selection marker, sense orientation; oen = neo selection marker, reverse orientation. References for the first publication of the mouse lines are given. FERM (F for 4.1 protein, E for ezrin, R for radixin and M for moesin), ANK (ankyrin repeat domain), SH3 (Src homology 3), PDZ (PSD-95/Discs large/zona occludens), SAM (sterile alpha motife). (Hung et al., 2008; Bozdagi et al., 2010; Peca et al., 2011; Wang et al., 2011, 2016; Schmeisser et al., 2012; Won et al., 2012; Kouser et al., 2013; Lee J. et al., 2015; Speed et al., 2015; Ha et al., 2016; Jaramillo et al., 2016, 2017; Mei et al., 2016; Peter et al., 2016; Zhou et al., 2016; Pappas et al., 2017).

This complex multidomain structure of the SHANK proteins and the diversity of SHANK1–3 isoforms might be one reason for the broad spectrum of *SHANK* variant-associated neurodevelopmental and neuropsychiatric disorders. Distinct *SHANK* mutations can yield different protein products, with a specific combination of protein domains, different from that of the WT protein. This, in turn, can affect the potential protein-protein interactions, thus altering the role of the protein in neurons. In ASD patients, chromosomal deletions, translocations, gene duplications, gene fragment deletions and coding mutations in all three *SHANK* genes were identified (see https://gene.sfari.org) but the simple haploinsufficiency caused by the mutation is unlikely to explain the broad spectrum of phenotypes. In order to dissect the causal link between *SHANK* variants and the underlying molecular mechanisms of neurodevelopmental and neuropsychiatric disorders, numerous mouse models for *“shankopathies”* were generated (Table 1, Figure 1). For SHANK3, 14 mouse lines, generated with different gene-targeted strategies, are published (Figure 1). Interestingly, the behavioral analysis of the *Shank3* knockout (KO) mouse lines revealed differences in social and repetitive behavior as well as in cognitive functions, similar to the distinct neuropsychiatric phenotypes of different patients. These behavioral inconsistencies were explained partially by the residual SHANK3 isoforms that could be expressed from the targeted *Shank3* gene in mice (Bozdagi et al., 2010; Peca et al., 2011; Yang et al., 2012) as discussed in detail in a recent review (Monteiro and Feng, 2017). However, in all 10 different *Shank2* KO mouse lines, the gene KO strategies were very similar leading to protein products truncated within the PDZ domain or the PRR, both present in all SHANK2 isoforms of the WT mice (Figure 1). Nevertheless, the phenotype of the three conventional and seven conditional *Shank2* KO mouse lines diverged between the different lines. This phenotypic variability is reminiscent of the fact that in human patients, *SHANK2* variants are not only significantly associated with ASD or ID, but also with schizophrenia (SCZ), a correlation that cannot be stated as strong for *SHANK3* variants (Peykov et al., 2015; Homann et al., 2016; de Sena Cortabitarte et al., 2017).

**Table 1:** Genetically modified mouse lines encoding gene-targeted mutations of endogenous *Shank* genes and one transgenic mouse line expressing a SHANK3-GFP that are made available to public in the mouse genome informatics database (http://www.informatics.jax.org).

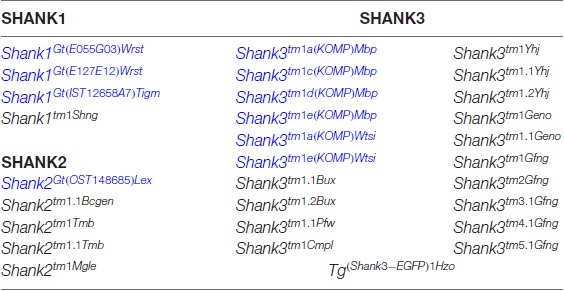

*Mouse lines in blue are not published but commercially available*.

## *Shank2* Gene Variants in Neurodevelopmental and Neuropsychiatric Disorders

After the first identification of *SHANK2* gene mutations in patients with ASD and ID (Berkel et al., 2010), several other publications described further variations in the *SHANK2* gene locus in patients with neuropsychiatric disorders. They identified *SHANK2* variations including truncations, missense mutations, gene deletions and mutations in the *SHANK2* promoter regions; all of these findings solidified the causal link of *SHANK2* variants to ASD and ID (Pinto et al., 2010; Wischmeijer et al., 2011; Leblond et al., 2012, 2014; Prasad et al., 2012; Chilian et al., 2013; Schluth-Bolard et al., 2013; Bowling et al., 2017; Yuen et al., 2017). Interestingly, an association between *SHANK2* gene mutations and SCZ was described in Peykov et al. (2015). By sequencing the *SHANK2* gene in 481 SCZ patients and 659 unaffected individuals, Peykov et al. (2015) identified several non-synonymous variants exclusively in SCZ patients (Supplementary Table S1). This association was confirmed by a study reporting seven siblings in a family with SCZ spectrum disorders carrying a missense variant in the *SHANK2* gene (Homann et al., 2016).

## How Divergent Phenotypes Can Arise From Different *SHANK2* Variants

### Mutations in Different Protein Interaction Domains

As outlined above, the comprehensive analysis of patients harboring *SHANK2* alterations with neurodevelopmental and neuropsychiatric disorders revealed a wide range of phenotypic expression, with various symptoms of ASD, ID, and SCZ. Moreover, the severity of *SHANK2*-associated disorders and cognitive deficiency is highly variable among patients. Imprecise clinical diagnoses and the clinical data from different clinics might explain this inconsistency. However, the *SHANK2* variants as such also need to be considered as one reason for such variability. Different mutations in the *SHANK2* gene can lead to increased or decreased expression of the SHANK2 protein, dysfunctional SHANK2 protein domains or truncated protein products lacking protein interaction domains. This can, in turn, alter protein-protein interactions and the organization of the postsynaptic protein network. Therefore, different *SHANK2* mutations can lead to different alterations in molecular and cellular processes in the neurons, resulting in a wide range of behavioral phenotypes.

This perspective finds support in the rescue experiment of SHANK2 knockdown by overexpression of human SHANK2 variants in rat primary cultures (Berkel et al., 2012). The SHANK2A(R462X) variant accumulated in the soma and the dendrites, whereas overexpressed SHANK2A-WT selectively located in dendritic spines (Berkel et al., 2012). The SHANK2A(T1127M) and SHANK2A(L1008_P1009dup) variants were confined into synapses with lower SHANK2A immunosignal compared to SHANK2A-WT (Berkel et al., 2012), indicating that various mutations can lead to a decrease in the total final amount of SHANK2A present at the synapse, which could be an important factor in the pathophysiology of the neuropsychiatric disorder. Furthermore, the dendritic spine volume was only significantly increased by the overexpression of SHANK2A-WT, whereas all three SHANK2A variants (R462X, T1127M, L1008_P1009dup) did not have the potential to increase the spine volume. Similarly, the SHANK2 knockdown, which provoked a reduced spine volume, an increased number of immature spines and a dendritic complexity near the cell body, was rescued to different degrees when SHANK2A variants were co-expressed with the SHANK2 knockdown shRNA in rat primary neurons thus suggesting a dosage effect in the case of SHANK2. The SHANK2A-WT and SHANK2A(L1008_P1009dup) were able to rescue the dendritic spine and the dendritic arbor development but SHANK2A(R462X) and SHANK2A(T1127M) could not rescue the reduced spine volume. Consistently, SHANK2A(R462X) failed to rescue the enhanced growth of dendrites to a normal level, whereas the SHANK2A(T1127M) and SHANK2A(L1008_P1009dup) were able to rescue the phenotype similar to the SHANK2A-WT. From these cell culture studies, it can be concluded that the *SHANK2* gene is involved in at least two separate functions: the control of dendrite morphology and the regulation of spine size.

### Genetic and Epigenetic Modulations

In addition to the nature of *SHANK2* variants, other genetic, epigenetic and environmental factors have a strong influence on the expression of the *SHANK2* variant-mediated neuropsychiatric disorders. For example, the same *SHANK2* variants were found in patients with different clinical features (Table 2). SHANK2(K535R) and SHANK2(P587S) variants were each identified in one ASD patient and in one patient with ID (Berkel et al., 2010). Similarly, SHANK2(A577V), previously misnamed as A578V, was associated with different characteristics of SCZ spectrum disorders in seven brothers (Homann et al., 2016). SHANK2(S610Y) was found in two patients: one with ID and the other with catatonic SCZ (Berkel et al., 2010; Peykov et al., 2015). The SHANK2(A1731S) variant was present in four patients with different subtypes of SCZ spectrum disorders (Peykov et al., 2015). In addition, some synonymous variants appear in patients with ASD, ID and SCZ, but not in healthy controls (Berkel et al., 2010; Rauch et al., 2012; Peykov et al., 2015; Supplementary Table S1). The influence of other genes on the phenotypic expression of the *SHANK2* variants became most obvious in patients with an inherited *SHANK2* variant-associated ASD or SCZ, which had not been diagnosed in their parents. The aforementioned SHANK2(A577V) variant found in seven schizophrenic male brothers, for example, was inherited from a healthy mother (Homann et al., 2016). This was also the case for five different SHANK2 variants: T438M, P1144L, V1608I, L1646M and A1731S (Peykov et al., 2015) and for several other variants identified in ASD patients (Supplementary Table S1) suggesting that sexually dimorphic pathways have an effect on the penetrance of these variants. Together, all these findings strongly support the proposed multiple hit model of neurodevelopmental and neuropsychiatric disorders (Leblond et al., 2012). Additionally, epigenetic factors most likely affect the penetrance of *SHANK2* variants. A DNA hypermethylation value of 5 CpG positions within the *SHANK2* gene was found in a male patient with ID and developmental delay (Kolarova et al., 2015), suggesting that *SHANK2* expression is sensitive to the DNA methylation pattern. Other epigenetic mechanisms such as histone acetylation are expected to regulate the expression of the *SHANK2* gene in an isoform-specific manner. Furthermore, one long non-coding variant associated to *SHANK2* has been found to be up-regulated in blood samples of ASD patients (Wang et al., 2015).

**Table 2:** Five single point mutations in the coding region (c.) of the *SHANK2* gene leading to five amino acid residue exchanges (p.) that are associated with different neuropsychiatric disorders.

Point mutation	Amino acid residue exchange	Number of patients	Diagnosis	Transmission source	References
c.1604A >G	p.K535R	2	1 ASD	n.a	Berkel et al. (2010)
			1 ID	n.a
c.1730C >T	p.A577V	7	5 SCZ	Mother	Homann et al. (2016)
			1 Schizotypal personality	Mother
			1 Schizoaffective	Mother
c.1759C >T	p.P587S	2	1 Autism	Mother	Berkel et al. (2010)
			1 ID	n.a
c.1829C >A	p.S610Y	2	1 Catatonic SCZ	n.a	Berkel et al. (2010)
			1 ID with Autistic features	Father	Peykov et al. (2015)
c.5191G >T	p.A1731S	4	3 Paranoid SCZ	Mother	Peykov et al. (2015)
			1 Disorganized SCZ	Mother	

*ASD, Autism spectrum disorder; ID, Intellectual disability; SCZ, Schizophrenia; n.a., not available. (NCBI reference sequence: NM_012309.4; for the full list of identified SHANK2 variants in patients, see supplementary information)*.

## Shank2 Isoforms in Mice

**Figure 2:**
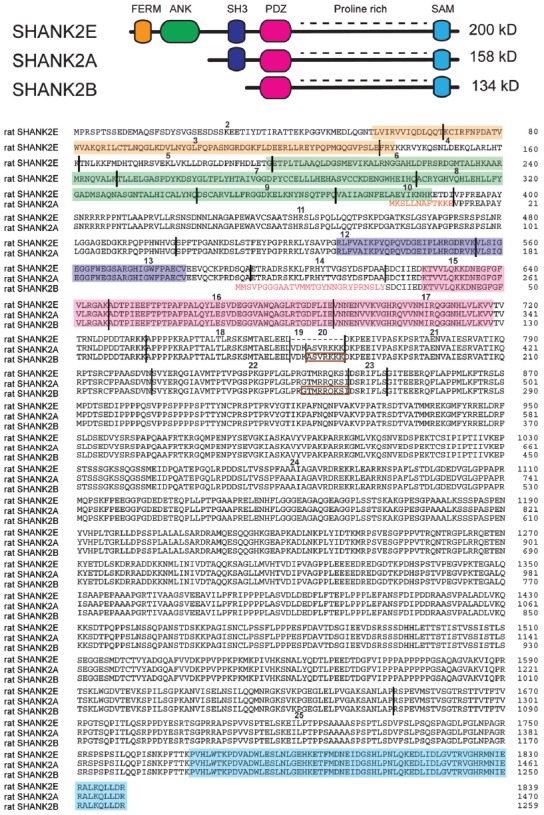
SHANK2 isoforms in the rat evaluated by direct cDNA cloning. (Top) Schematic view of different conserved protein domains in SHANK2 isoforms. (Bottom) Amino acid residue sequence alignment of the SHANK2E (AY298755; Han et al., 2006), SHANK2A (AJ249562; Boeckers et al., 1999a) and SHANK2B (AF060116 or AJ131899; Du et al., 1998; Boeckers et al., 1999b) isoforms. (Bottom) Amino acid residue sequence alignment of the SHANK2E (AY298755; Han et al., 2006), SHANK2A (AJ249562; Boeckers et al., 1999a) and SHANK2B (AF060116 or AJ131899; Du et al., 1998; Boeckers et al., 1999b) isoforms. The SHANK2 protein domains are color-coded. The positions of the introns in the coding regions are indicated by vertical black lines. Exons are numbered starting with exon 2 as the first translated exon. N-terminal amino acid residues of isoform A and B are given in red. The sequences inside the brown rectangles are absent in some other *Shank2* transcripts. The N-terminal sequence of isoform A is encoded by the three terminal end of intron 10. The N-terminal sequence of isoform B is encoded in intron 14. Isoform C which is found in humans cannot be detected in mouse or rat mRNAs.

For the design and the possible impact of *Shank2* gene manipulation in mice, the detailed knowledge of the different SHANK2 isoforms and their expression pattern in the mouse brain is a prerequisite. As *Shank1*–*3* can express many different SHANK isoforms using several promoters and alternative splicing (Figure 1), the different SHANK isoforms are proposed to have different functions during developmental stages (Jiang and Ehlers, 2013). According to the NCBI database, 21 putative isoforms are predicted to be expressed by the *Shank2* gene locus on chromosome 7 in mice. Cloned rat cDNAs for the SHANK2 isoforms, SHANK2E, SHANK2A and SHANK2B, provide experimental evidence that these three isoforms are differentially expressed in the rat brain (Figure 2; Boeckers et al., 1999a,b; Han et al., 2006). Three different promoters are recruited for the expression of SHANK2E, SHANK2A and SHANK2B. The 5’ located promoter is used to generate the largest isoform, SHANK2E. The SHANK2E encoding transcript contains a 5’-untranslated exon, which is designated as exon 1. The promoters for the initiation of *Shank2a* and *Shank2b* transcripts are located in introns 10 and 14, respectively. For the translation of *Shank2a* and *Shank2b* transcripts, intron-located translational start codons open the translational reading frames (Figure 2).

The mouse *Shank2* gene contains 25 exons. The alternative splicing of exon 19, 20 and 23 and the internal alternative splice donor site in exon 22 can lead to additional isoforms of SHANK2E, SHANK2A and SHANK2B. Using rat brain and human brain-derived cDNAs, the presence and absence of the alternatively spliced *Shank2a* exons were experimentally verified in rats and humans. The transcripts with exons 19, 20 and 23 seem to be underrepresented in peripheral tissues, whereas they seem to be dominantly expressed in the brain (Böckers et al., 2004; Leblond et al., 2012). It is important to mention that the short SHANK2C isoform, which is obtained by an alternative splicing event of exon 22 in humans (Leblond et al., 2012), cannot be detected in the transcriptom of mice and rats. The description of the presence or absence of exon 19 and 20 in the NCBI database-predicted 21 SHANK2 isoforms in mice is inconsistent and needs to be verified. This might explain why different numbers of exons in the *Shank2* gene loci are given in different genome browsers (e.g., in Ensemble: ENSMUSG00000037541, the mouse *Shank2* gene is composed of 23 exons).

As shown in Figure 2, SHANK2E is the full-length SHANK2 isoform containing an ANK domain, SH3 domain, PDZ domain, PRR and SAM domain, which is located on the SHANK2 C-terminal synaptic targeting elements (Böckers et al., 2004; Boeckers et al., 2005). In the brain, SHANK2E is expressed at different levels in different regions with the highest expression in the cerebellum (Leblond et al., 2012). The shortest SHANK2 isoform, SHANK2B (named as SHANK2C in Monteiro and Feng, 2017), contains just the PDZ, PRR and SAM domains (Figure 2). As determined by mRNA analysis in humans and rats, *SHANK2* expression is strongest in the brain, but *SHANK2* mRNA can also be PCR-amplified in most peripheral tissues with the exception of skeletal muscles and heart (Leblond et al., 2012). The *SHANK2B* RT-PCR products confirmed the differential *SHANK2B* expression in all human brain regions (Leblond et al., 2012), a finding that had already been well documented in rats by classical *in situ* RNA hybridization studies showing brain region-specific as well as developmentally controlled expression of SHANK1–3 (Böckers et al., 2004). According to this study, SHANK2 is co-expressed with SHANK1 during the early days of development, whereas SHANK3 reaches its maximum expression at postnatal day P16. By using the laminar organization of the cerebellum and the hippocampus, the authors showed convincingly that: (i) *Shank1* and *Shank2* mRNA co-exist in Purkinje cells (PCs) and their dendrites, whereas *Shank3* mRNA was found only in the cerebellar granular cells; and (ii) *Shank2* mRNA expression in the hippocampus is restricted to the cell layer of principal neurons, while the signals for the *Shank1* and *Shank3* mRNA can be detected in the molecular layers as well (Böckers et al., 2004).

In biochemical studies using rats, SHANK2 and SHANK3 seemed to be essential elements for the proper organization of the PSD. They localize to the PSD via their SAM domain and interactions with Zn^2+^ ions, which can be enhanced by the activity and synaptic release of Zn^2+^. In contrast, SHANK1 is recruited to the PSD by a pre-formed scaffold via its PDZ domain (Baron et al., 2006; Gundelfinger et al., 2006; Grabrucker et al., 2011). Thus, all SHANKs can respond to activity and synaptic events that might underlie learning and memory. However, SHANK1 is lacking the capability to localize to immature/inactive synapses, which seems to be SAM domain/Zn^2+^-dependent (Schmeisser and Verpelli, 2016). During neuronal development, the subcellular distribution of SHANK2 proteins changes and immunoelectron microscopy studies revealed that SHANK2 accumulation shifts in the developing, cortical nuerons. At postnatal day P5, SHANK is accumulated in the lamellipodium, then shifts into the cytoplasm of the cell bodies and in the growing neurites at P8, and finally to the PSD at P10, which supports the idea that it may play a role in multiple cell biological frameworks of neurons (Boeckers et al., 1999a). Interestingly, on the electrophysiological level, SHANK1 and SHANK2, but not SHANK3 virus-mediated knockdown reduced the number of spines and the AMPAR responses at CA3-to-CA1 synapses in acute hippocampal slice cultures (Shi et al., 2017). Due to this functional divergence of SHANK proteins, it is not surprising that in ASD patients, *SHANK2* and *SHANK3* mutations are over-represented (Leblond et al., 2014).

## Modeling *Shank2* Mutations in Mice

The multiple genetic, epigenetic and environmental factors affecting the phenotypic expression of ASD and SCZ disorders are a major issue when we try to understand the molecular, anatomical, physiological and behavioral phenotypes of *SHANK2* variants. Analyzing genetically comparable inbred mice should at least minimize the environmental and epigenetic components when mice are raised and housed under comparable conditions. Observations of male and female single-housed or cohort-housed mice with identical or mixed genotypes, and either with conditional gene deletions or gene overexpression, offer multiple options to investigate different aspects of the neurodevelopmental and neuropsychiatric disorders in an animal model. Already in 2007, Crawley and Tordjman proposed several behavioral tasks that are suitable for the analysis of autistic behavior in mice (Crawley, 2007; Tordjman et al., 2007).

### Distinct Phenotypes of the Different *Shank2* Knockout Mice

The proposal of Crawley and Tordjman likely promoted the generation of several gene-targeted mutations in mice modeling human *SHANK1, 2* and *3* mutations-associated ASD (Figure 1). In 2012, the first two *Shank2* KO mouse models (*Shank2^−/−^*) were generated. Both mouse models resembled two PDZ domain-microdeletions found in two human patients (Berkel et al., 2010). Mice of both lines showed comparable cognitive and social impairments. In *Shank2^Δex16^* mice (named *Shank2^Δex7^*), exon 16 was deleted (Schmeisser et al., 2012). In *Shank2^Δex15-16^* mice (named *Shank2^Δex6-7^*), the exons 15-16 encoding gene segment was replaced by a loxP site flanked inverse-oriented neomycin resistance (neo) selection marker (Won et al., 2012). Although no residual SHANK2 expression was detected in homozygous *Shank2*^Δex*16*^ or *Shank2*^Δex*15-16*^ mice as determined by immunoblots and immunohistology, and although the genetic background of the two mouse lines was similar (C57Bl/6N and C57Bl/6J), significant differences on the molecular, electrophysiological, synaptic composition and anatomical levels of *Shank2*^Δex*16*^ or *Shank2*^Δex*15-16*^ were evident (Table 3, Supplementary Table S2). The impairment of synaptic plasticity at hippocampal CA1 synapses showed opposite results in the two mouse lines and the NMDAR response was increased in *Shank2^Δex16^* but decreased in *Shank2*^Δex*15–16*^ mice. In addition, the spine number and spine density were reduced in CA1 pyramidal cells of *Shank2^Δex16^*, whereas *Shank2^Δex15-16^* mice showed normal spine numbers and density (Table 3, Supplementary Table S2).

**Table 3:** Distinct endophenotypes in genetically very similar gene-manipulated, conventional *Shank2* knockout mice.

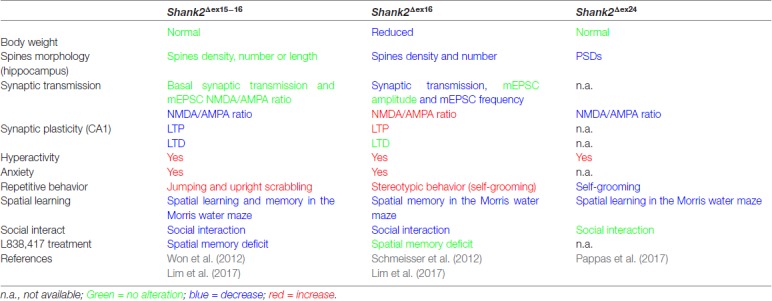

**Figure 3:**
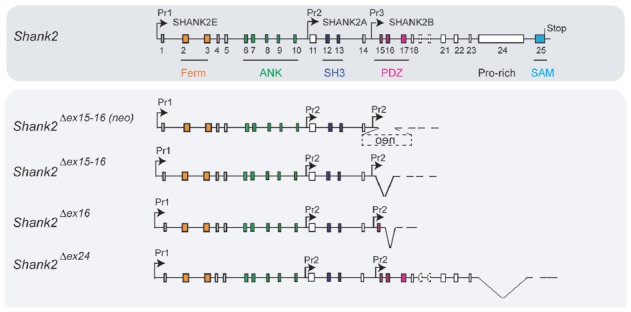
Putative pre-mRNA transcripts in homozygous *Shank2* KO mice. Schematic view of the *Shank2* gene and the putative pre-mRNA that can be expressed by the four different gene-targeted alleles of the *Shank2* mouse models with very distinct phenotypes. Symbols are as in Figure 2.

In 2017, Lim et al. (2017) substantiated the distinct phenotypes of *Shank2^Δex16^* and *Shank2^Δex15-16^* mice by a direct comparison of the two mouse lines in a mixed C57Bl/6N × C57Bl/6J background. The differences in gene expression (Supplementary Table S2) and synaptic properties such as the AMPA/NMDA ratio and long-term potentiation (LTP; Neves et al., 2008) as well as differences in the inhibitory signaling were directly correlated to the *Shank2* Δex*16* and Δex*15-16* gene-targeted mutations. The *Shank2^Δex15-16^* mice exhibited a decreased GABA_A_R-mediated inhibition, most likely caused by the reduced expression of the GABAR gene, *Gabra2*. Interestingly, a similar discrepancy between the phenotypic expression of *Shank2* Δex*16* and Δex*15-16* gene deletions was found when the two gene deletions were restricted to cerebellar PCs (Ha et al., 2016; Peter et al., 2016; Supplementary Table S2). These mouse lines were generated by combining the floxed *Shank2 ex16* or the floxed *Shank2 ex15-16* gene, with transgenic Cre-expressing mouse lines that used the PC specific Pcp2 promoter, also called L7 promoter, for PC specific inactivation of the *Shank2* gene (Figure 1). *Shank2^Δex15-16–Pcp2-Cre^* mice showed impaired motor coordination and learning in the Erasmus ladder test, but normal motor performance in the rotarod test. However, they exhibited neither repetitive behavior nor social interaction deficits, but only a mild anxiety-like behavior. In contrast, *Shank2^Δex16–L7–Cre^* mice showed normal motor performance in the Erasmus ladder with no anxiety-like behavior, but deficits in social interaction as well as social novelty recognition. Thus, similar inactivation of the *Shank2* gene in cerebellar PCs produced distinct phenotypes at the molecular and up to the behavioral level. Similarly in two recent mouse models restricting the Δex*15-16* deletion in two specific cell types; one in excitatory neurons (*Shank2^Δex15-16–CaMK2a-Cre^*) and the other in GABAergic inhibitory neurons (*Shank2^Δex15-16–Viaat-Cre^*, Kim et al., 2018), *Shank2^Δex15-16–CaMK2a-Cr^* and *Shank2^Δex15-16–Viaat-Cre^* mice showed differences on the electrophysiological level by reduced mEPSC frequency in hippocampal CA1 neurons in *Shank2^Δex15-16–CaMK2a-Cre^* but not in *Shank2^Δex15-16–Viaat-Cre^* mice. *Shank2^Δex15-16–CaMK2a-Cre^* mice showed increased anxiety-like but normal repetitive behaviors in comparison to *Shank2^Δex15-16–Viaat-Cre^* mice, which showed no anxiety-like but increased repetitive behaviors (Supplementary Table S2). Thus, in *Shank2* KO mice, a residual expression of truncated proteins or mRNAs, which differs between the *Shank2* mouse lines (Figure 3), might disturb the structure or the flexibility of the SHANK scaffold at different levels and thus the flexibility of the postsynaptic protein organization. This impaired synaptic function might vary in different neurons or even be different in various synapses of the same neuron. These finding in cell type specific *Shank2* KO mice underlines observations that very similar *SHANK2* mutations can lead to a different phenotype, as noticed by the wide behavioral spectrum of ASD described for patients with mutations in the *SHANK2* genes (Supplementary Table S1).

In 2017, a third conventional *Shank2* KO mouse was generated by the out-of-frame deletion of *Shank2* exon 24, which deleted the PRR region (Pappas et al., 2017). These *Shank2^Δex24^* KO mice exhibited a pronounced behavioral difference compared to the published ASD-like behavior of *Shank2*^Δ*ex16*^ and *Shank2^Δex15-16^* (Table 3, Supplementary Table S2). *Shank2^Δex24^* mice showed a bipolar-associated mania-like behavior. *Shank2^Δex24^* mice were hyperactive in the home cage and open field test, showed a decrease in repetitive behaviors such as self-grooming, had no social preference in the social affiliation test nor did they show a SCZ-like behavior. Instead, *Shank2^Δex24^* mice showed anhedonia-like behavior and disturbed circadian rhythms. Some of these unique behavioral impairments persisted even in conditional *Shank2^Δex24^* mice (Supplementary Table S2; Pappas et al., 2017). When exon 24 of *Shank2* was removed in cerebellar PCs, *Shank2^Δex24-Pcp2-Cre^* mice showed an impaired motor performance in the rotarod (Pappas et al., 2017). The Cre-mediated removal of exon 24 in the neocortex in *Shank2^Δex24-Emx1-Cre^* was correlated with mania-like behavior, hyperactivity, impaired spatial memory and a surprisingly significant social preference in female mice (Pappas et al., 2017). The developmental contribution of the *Shank2^Δex24^* was unraveled by the conditional deletion of exon 24 in forebrain postnatally, using the developmentally-controlled promoter for the alpha subunit of Ca^2+^-calmodulin dependent protein kinase II (CaMKII) for Cre expression. Thus, when exon 24 was deleted efficiently in 3 weeks old *Shank2^Δex24-CaMKI2a-Cre^* mice, they didn’t show any hyperactivity, which was used as signature for the mania-like behavior of the global *Shank2^Δex24^* mice (Pappas et al., 2017).

### How Can the Distinct Phenotypes of the Different *Shank2* Knockout Mice Be Explained?

The heterogeneity in the phenotypic expression of all three conventional and seven conditional *Shank2*-“deficient” mouse models indicated that there is no clearly defined SHANK2 deficiency-mediated phenotype. Similar to human patients, each *Shank2* KO mouse line showed a distinct neuropsychiatric-like phenotype, which was dependent on the specific type of *Shank2* mutation. In contrast to humans, where genetic and environmental factors cannot be excluded as cofactors for the penetrance of abnormal social behavior, the common social experience of experimental mouse cohorts can largely exclude environmental factors. Therefore, it is more likely that similar genetic modulations still express different truncated SHANK2 proteins at different levels (Figure 3), which might disturb the balanced co-expression of both SHANK1 and SHANK3 isoforms and exert more different dominant-negative effects on other protein interacting partners, that can be recognized by different behavioral outcomes. Experimental evidence for disturbed or unbalanced expression of SHANK proteins was provided by the virus-mediated overexpression in mice of the truncated SHANK2A(R462X) variant (Berkel et al., 2012), initially identified in one ASD patient (Berkel et al., 2010; Supplementary Table S2). Neurons in hippocampal cultures showed impaired spine development, characterized by their filopodia-like structure. In CA1 cells from acute hippocampal slices, reduced mEPSC amplitudes could be recorded which were correlated to impaired cognitive function, demonstrating that the truncated SHANK2 proteins can disturb the physiological function of the coordinated SHANK expression in the CNS.

Thus, as already described for similar *Shank3* KO mouse models, the *Shank2* KO lines are characterized by functionally distinct molecular, electrophysiological and neurophysiological phenotypes. In particular, the opposing effect of the disturbed excitatory and inhibitory responses of CA1 pyramidal cells in *Shank2^Δex15-16^* and *Shank2^Δex16^* was unexpected and still is not well understood. Already small differences in the genetic background could be responsible for this drastic difference. Moreover, it might be interesting to find out whether female and male mice or littermates with the very same *Shank2* KO mutation show different neuropsychiatric-like behavior, as described in human patients for four and seven siblings, who inherited the *SHANK2* mutation from their healthy mothers (Table 2). The careful comparative analysis of individual *Shank2* KO littermates of the same KO mouse line might identify epigenetic and environmental ASD or SCZ-facilitating factors; although this is a major and long-lasting endeavor.

## Insight Into ASD Pathogenesis and Treatment From *Shank2* Knockout Mouse Models

The finding of distinct phenotypes of genetically very similar *Shank2* KO mice finds further strong support by the pharmacological treatment strategies of the different *Shank2* KO mice. In the *Shank2^Δex15-16^* mice, the hippocampal NMDAR function in brain slices was decreased (Won et al., 2012). The treatment of *Shank2^Δex15-16^* with D-cycloserine, a partial agonist at the glycine-binding site of NMDARs, increased the NMDAR responses and rescued the social impairments of *Shank2^Δex15-16^* mice. Similarly, the administration of a positive allosteric modulator of mGluR5, 3-cyano-N-(1,3-diphenyl-1H-pyrazol-5-yl benzamide (CDPPB), which enhances the NMDAR function via mGluR5 activation, rescued the NMDAR signaling impairments, as measured in hippocampal CA1 cells. The CDPPB-mediated recovery of NMDAR function in *Shank2^Δex15-16^* mice was correlated with normalized social interaction of CDPPB treated *Shank2^Δex15-16^* mice. However, hyperactivity and anxiety-like behaviors were not recovered (Won et al., 2012).

A different approach to activate the NMDAR function in *Shank2^Δex15-16^* mice was carried out by inducing postsynaptic Zn^2+^ elevation using a Zn^2+^ chelator, clioquinol (Lee E. J. et al., 2015). Zn^2+^ is mainly derived from presynaptic pools and activates NMDAR through postsynaptic activation of the tyrosine kinase Src (Manzerra et al., 2001). Treating *Shank2^Δex15-16^* mice with clioquinol enhanced social interaction, but did not had any effect on social novelty recognition, anxiety-like behavior, hyperactivity and repetitive behavior. The functional NMDAR rescue was demonstrated by restoring NMDAR signaling at hippocampal (SC-CA1) synapses (Lee E. J. et al., 2015). Although the pharmacological treatment of *Shank2^Δex15-16^* mice was carried out 30–120 min before the behavioral tests, and even though the recovery of the NMDAR function was shown at hippocampal synapses, other brain regions, e.g., the oxytocin neurons in the hypothalamus, might also be involved in producing social incompetence and could be good candidate targets for pharmacological therapy.

In addition to the stimulation of the glutamatergic system, the enhancement of the inhibitory synaptic activity can reverse genetically-based ASD-associated deficits. In *Shank2^Δex15-16^* mice, *Gabra2* expression on RNA level as well as GABA_A_ receptor-mediated inhibitory neurotransmission in CA1 hippocampal slices were reduced in *Shank2^Δex15-16^*, but not in *Shank2^Δex16^* mice. Treatment with an allosteric modulator of GABA_A_ receptor, L838,417, reversed the spatial memory deficits of the *Shank2^Δex15-16^* mice, but not those of *Shank2^Δex16^* mice (Lim et al., 2017). However, L838,417 had no significant effects on anxiety-like behavior or social impairment in *Shank2^Δex15-16^* mice. Whether or not long or short-term treatment in young and adult mice will have a persistent rescue effect remains to be investigated. The pharmacological rescues showed that the identification of the underlying electrophysiological mechanism for the type of ASD is mandatory for a successful treatment. Since a detailed electrophysiological differential diagnosis of ASD patients is not feasible, the *Shank* mutant mice are the best accessible tools to dissect different ASD endophenotypes of *SHANK* mutations at the molecular, anatomical, electrophysiological and behavioral levels.

## Behavioral Test Battery for *Shank2* Mouse Models

The widely varying phenotypes of *Shank2* mouse models for ASD and other neuropsychiatric disorders can be grouped into several categories: molecular, anatomical, hormonal, immunological, neurophysiological and behavioral. The detailed description within all six categories is crucial for the putative therapeutic treatment which, if successful, will likely to alleviate only some of ASD, SCZ or cognitive phenotypes. Standardized molecular, anatomical and neuronal activity analyses are straightforward and comparable between different studies, at least when the analyzed mutant and control mice are derived from the same colony, are age-matched, of the same gender, and when the genetic background of the mice used in different studies is similar. A major issue is comparing behavioral phenotypes determined in different laboratories (Schellinck et al., 2010), which can be a reason for the difference in behavior between the *Shank2* mouse models.

For the comparable description of ASD and other neuropsychiatric disorder phenotypes in *Shank2* mouse models, it is obligatory to exclude gross physical abnormalities (Crawley, 2007). General health, body weight and neurological reflexes including eye blink, ear reflex and whisker twitch should be investigated. The three-stage SHIRPA procedure (Immelmann and Beer, 1989) can be performed to assess mice phenotypes for obvious physical, behavioral and morphological impairments before testing social behavior and cognition. In the best case, a complete ethogram of the *Shank2* mutant mouse line, before and after genetic or pharmacological rescue, should be determined to cover the expected wide spectrum of phenotypes. If only a partial ethogram is provided, at least the full range of social and cognitive performance tests should be presented. As suggested (Crawley, 2007; Tordjman et al., 2007), a consistent “multi-trait” analysis of cognitive and social behavior is necessary for the dissection of the clinically heterogeneous ASD spectrum in the different mouse models.

### First Simple Behavioral Tests

For *Shank2* mouse models, the first behavioral observations can already be started when the mouse is still used to live in its home cage. The burrowing or marble burying tests are indicators for anxiety and obsessive-compulsive disorders (Broekkamp et al., 1986; Borsini et al., 2002; Deacon, 2006; Thomas et al., 2009; Angoa-Pérez et al., 2013). The nest building test (Deacon, 2012) is considered a potential endophenotype relevant to the negative symptoms of SCZ (Amann et al., 2010) and depression. The unbiased physical performance and activity, as well as circadian rhythm of mice, can be recorded in the home cage (LABORAS system from Metris) and the running wheel; motor coordination in rotarod and Erasmus ladder test (Dunham and Miya, 1957; Van Der Giessen et al., 2008; Vandeputte et al., 2010; Luong et al., 2011). For anxiety testing, which is considered one of the most important co-morbidities of neuropsychiatric disorders, the zero-maze, elevated zero-maze and elevated plus-maze (Handley and Mithani, 1984; Pellow et al., 1985; Lister, 1987; Shepherd et al., 1994; Heisler et al., 1998; Cook et al., 2001) and the dark-light compartment (Crawley and Goodwin, 1980) can be used.

### The Four Most Important Diagnostic Symptoms Need to Be Investigated

The behavioral test battery of *Shank2* mouse models should focus primarily on diagnostic symptoms of ASD: abnormal social interactions, deficits in communication, high levels of repetitive behaviors and cognitive dysfunction. However, to express cognitive and social competence in a behavioral test, anxiety and other stress factors have to be minimized. Pre-handling of the mice for at least 1 week is obligatory before testing the social behavior and memory (Fridgeirsdottir et al., 2014). In addition, increased stress by different housing conditions might affect the results (Kamakura et al., 2016). During the handling phase, repetitive behaviors (self-grooming, jumping and climbing) can be recorded in the LABORAS, and repetitive digging behavior in the marble burying assay. Social ability is usually assessed in the three-chambered box (Moy et al., 2004), the partition test apparatus (Kudryavtseva, 2003) and reciprocal social interaction in a novel arena (Silverman et al., 2010). When communicating information, olfactory stimulations as well as vocalizations in the ultrasonic ranges are used to enhance social bonding between mice, which can be evaluated by the olfactory habituation/dishabituation test (Wesson et al., 2008) and by measuring ultrasonic vocalizations (USV). As a measure of cognitive function, the spatial working reference memory can be tested in the T-maze and spatial reference memory in the Morris water maze, Y- or Radial-maze. The simple puzzle box is testing the goal-oriented performance of mice, which can give early hints of cognitive functions (Ben Abdallah et al., 2011). Social transmission of food preference has been used as a method for studying memory by several laboratories (Wrenn, 2004).

### Additional Behavioral Assays Analogous to Other Symptoms of Neuropsychiatric Disorders

Patients with neuropsychiatric disorders suffer from a wide range of symptoms which can be measured in *Shank2* mice to some extent. Depression in mice can be noticed in the forced swim test, the tail-suspension tests (Porsolt et al., 1977; Steru et al., 1985) and by anhedonia in the sucrose preference test (Papp et al., 1991; Tye et al., 2013). Deficits in sensory processing can be considered a symptom of SCZ, which can be evaluated by the Pre-Pulse Inhibition (PPI) of Acoustic Startle Response (ASR; Braff and Geyer, 1990; Swerdlow et al., 1994). Other symptoms of SCZ, such as delusions and auditory hallucinations, still cannot be fully assessed in mice (Chadman et al., 2009). If necessary, learning and memorizing fear can be examined as the last step in the test battery. However, the analysis of contextual and cued fear learning (Maren, 2001) or the passive avoidance test (Gray and McNaughton, 2000) are usually not performed with *Shank2* mouse models (for the summary of tests see Table 4).

**Table 4:** Summary of behavioral tests that can be used in the test battery for *Shank2* mouse models and other mice with potential neuropsychiatric-like phenotype.

Targeted domain	Behavioral tests	Targeted domain	Behavioral tests
**General**	- SHIRPA	**Mania**	- Circadian rhythm in running wheel - Videotaped observations of home cage sleep and activity patterns
**Hyper/hypo activity**	- Locomotor activity in the home cage - Locomotor activity in the open field	**Seizure**	- Sensitivity to audiogenic seizures - Sensitivity to drug-induced seizures
**Anxiety**	- Elevated plus-maze - Elevated zero-maze - Dark-light compartment - Open field - Marble burying - Shock-probe burying - Vogel conflict test - Hyponeophagia	**Depression**	- Forced swim test - Tail-suspension test - Visible Burrow System - Learned helplessness test - Sucrose preference task - Circadian rhythm - Social interaction - Sexual behavior
**Motor learning, balance, coordination and impulsivity**	- Rotarod - Running wheel - Open field - Horizontal bar - Static rods - Parallel bars - Mouse cylinder test - Elevated bridge - Swim test - Staircase test - Hanging wire test - Hind-paw footprint test - Balance beam test - Erasmus ladder test - Eye-blink conditioning test - Cliff avoidance test - 5-Choice serial reaction time task - Stop-signal reaction time task	**Cognitive, attention and memory function**	- Novel object recognition task - Morris water maze - Fear conditioning - Pre-pulse inhibition - Mazes (Barnes, Radial, Y and T) - Hole-board - Odorant-based tasks - Operant conditioning - 5-Choice serial reaction time task - Set-shifting task - Latent inhibition test - Social transmission of food preference task - Eye-blink conditioning
		**Response to sensory stimuli**	- Acoustic startle - Tactile startle - Hot plate
**Repetitive behavior, resistance to change in routine**	- Hole-board - Marble burying - Reversal learning in the Morris water maze or Y-maze - Self-grooming - Motor stereotypies (rearing and jumping)	**Aggression**	- Von Frey hairs - Attentional neglect tape test - Resident-intruder test - Isolation-induced fighting - Tube test
**Social interaction, communication and social dominance**	- 3-chamber social test - Direct social interaction - Social conditioned place preference task - Operant conditioning - Partition test - Visible Burrow System - Juvenile play - Whisker trimming - Nest building - USV during social interaction - USV by separated pups - Retrieval of separated pups - Olfactory habituation/dishabituation measures - Tube test	

## Conclusion

The different alterations in synaptic responses, synaptic plasticity, social interaction and exploration behavior, as well as therapeutic responses in *Shank2* KO mice emphasize that a balanced, physiologically-controlled expression of SHANKs is necessary for the appropriate organization of postsynaptic proteins and receptors at excitatory synapses. In addition, the comparison between *Shank2* KO mouse lines strongly suggests that truncated SHANK2 isoforms or their mRNAs disrupt the homeostasis of SHANK proteins and their function as major scaffolding organizers in the postsynaptic matrix. Different *Shank2* mutations might yield different protein products with potentially contrasting effects at the synapse (e.g., increasing or decreasing its strength), thus leading to a diversity of phenotypes. However, epigenetic factors as well as inconsistent analysis of the various *Shank2* mouse lines cannot yet be completely ruled out.

## Perspective

Understanding the detailed function of the multitude of SHANK isoforms is one of the biggest challenges in cognitive neuroscience. The disturbed balance of different SHANK proteins within individual postsynapses significantly affects the plasticity of synapses, possibly by changing the composition of the postsynaptic scaffold, which translates to a rearrangement of the glutamate receptors and measurable alterations in the glutamatergic signaling. For *in vivo* analysis of certain SHANK2 isoforms in mice, certain exons or *SHANK* internal promoters could be eliminated or certain SHANK isoforms could be overexpressed by viruses or transgenes. New genetic engineering techniques can generate relatively quickly novel *Shank2* mouse lines. However, in future studies with novel constitutive and conditional *Shank2* mouse lines, the expression of the residual truncated isoforms should be analyzed in more details, and the full battery of behavioral analysis covering ASD, SCZ, mania and Attention Deficit Hyperactivity Disorder (ADHD) has to be applied, since the existing *Shank2* mouse models showed very distinct, unpredictable phenotype despite very similar genetic manipulations. In addition, males and females and real littermates need to be compared in detail to unravel epigenetic, environmental and gender effects on the expression of the phenotypes. More importantly, the current *Shank2* mouse models provided first insights into molecular and physiological changes underlying ASD-related symptoms. The *Shank2* mutant mice showed that ASD-related symptoms can be diminished by pharmacological treatment of the mutant mice, which can guide future therapeutic strategies for ASD patients. Now the application of spatio-temporal removal of SHANK2 using cell-type specific Cre or CreERT2 mice can identify those neuronal connections and circuits that have the strongest impact on the expression of the ASD-like phenotype. Moreover, conditional *Shank2* KO mice can help to dissect the neurodevelopmental vs. the transient deficiencies mediated by the SHANK depletion. Once the most crucial neuronal circuits and cell types are identified, those neuronal populations can be targeted and analyzed in great detail in behaving mice by novel physiological technologies, e.g., multicellular recordings and optogenetics, which allow the recording, visualization and manipulation of neuronal activity ensembles during social interaction, repetitive behavior, vocalization and cognition, or even during different phases of sleep. Due to the very distinct phenotypes of different *Shank2* mouse lines, it will be exciting to see how their altered neuronal network activity is correlated with the impaired social behavior.

## Author Contributions

All authors significantly contributed to the manuscript. AE and RS wrote the first draft and provided the figures, tables and supplementary information. GR critically revised the manuscript.

## Conflict of Interest Statement

The authors declare that the research was conducted in the absence of any commercial or financial relationships that could be construed as a potential conflict of interest.
