# Supplementary material for: Distinct Phenotypes of Shank2 Mouse Models Reflect Neuropsychiatric Spectrum Disorders of Human Patients With SHANK2 Variants
Source: Front Mol Neurosci. 2018 Jul 19;11:240. doi: 10.3389/fnmol.2018.00240 (PMC6060255; doi:10.3389/fnmol.2018.00240)
Supplement: Supplementary file 1 [file Table_1.pdf]

Supplementary Material

Distinct Phenotypes of *Shank2* mouse models reflect neuropsychiatric spectrum disorders of human patients with *SHANK2* variants

Ahmed Eltokhi, Gudrun Rappold, Rolf Sprengel*

*** Correspondence:** Rolf Sprengel: Rolf.Sprengel@mpimf-heidelberg.mpg.de

Supplementary Table 1a and b: Summary of *SHANK2* gene variants in the coding sequence identified in patients with neurodevelopmental and neuropsychiatric disorders but not in healthy human controls. (NCBI reference sequence: NM_012309.4). Data from Table 2 are included and highlighted in grey.

Supplementary Table 2 a,b,c and d: Phenotypes of different global and conditional *Shank2* knockout mice and of mice with rAAV-mediated overexpression of truncated SHANK2A isoform.

Supplementary Table 1a

Mutation	Affected amino acid	Variant type	Number of patients	Phenotype	Sex	Transmission source	Reference
c.132G>A	p.P44P	synonymous	1	ID	F	*de novo*	(Rauch et al., 2012)
c.554G>A	p.R185Q	missense	1	Autism	M	Mother	(Leblond et al., 2012)
c.1213C>T	p.R405W	missense	1	Autism	F	Father	(Berkel et al., 2010)
c.1313C>T	p.T438M	missense	1	SCZ	M	Mother	Peykov et al., 2015
c.1327C>T	p.R443C	missense	1	Autism with ID	M	Father	(Leblond et al., 2012)
c.1463 G>T	p.G488V	missense	1	SCZ	F	n.a.	(Peykov et al., 2015)
c.1575C>T	p.A525A	synonymous	1	ID	n.a.	n.a.	(Berkel et al., 2010)
c.1604A>G	p.K535R	missense	2	1 ASD<br>1 ID	n.a.<br>n.a.	n.a.<br>n.a.	(Berkel et al., 2010)
c.1629C>T	p.G543G	synonymous	3	3 ASD	n.a.	n.a.	(Berkel et al., 2010)
c.1730C>T	p.A577V	missense	7	5 SCZ,<br>1 schizotypal personality,<br>1 schizoaffective	7 M	Mother	(Homann et al., 2016)
c.1759C>T	p.P587S	missense	2	1 Autism<br>1 ID	M<br>n.a.	Mother<br>n.a.	(Berkel et al., 2010)
c.1793G>T	p.R598L	missense	1	Autism with ID	M	n.a.	(Leblond et al., 2012)
c.1829C>A	p.S610Y	missense	2	Catatonic SCZ<br><br>ID with some autistic features	F<br><br>F	n.a.<br><br>Father	(Peykov et al., 2015)<br><br>(Berkel et al., 2010)
c.1845C>T	p.D615D	synonymous	1	SCZ	n.a.	n.a.	(Peykov et al., 2015)
c.1896dupA	p.D633Rfs	frameshift	1	ID with ADHD	n.a.	*de novo*	(Bowling et al., 2017)
c.2069A>G	p.N690S	missense	1	Disorganized SCZ	F	n.a.	(Peykov et al., 2015)
c.2149G>T	p.V717F	missense	1	Autism	M	Father	(Leblond et al., 2012)
c.2185G>A	p.A729T	missense	1	Autism with ID	F	Mother	(Leblond et al., 2012)
c.2421G>A	p.P807P	synonymous	1	ASD	n.a.	n.a.	(Berkel et al., 2010)
c.2439C>T	P.N813N	synonymous	1	SCZ	n.a.	n.a.	(Peykov et al., 2015)
c.2521C>T	p.R841X	stop_gained	1	ASD with ID	M	*de novo*	(Berkel et al., 2010)<br>(Yuen et al., 2016)
c.2529G>T	p.T843T	synonymous	1	ASD	n.a.	n.a.	(Berkel et al., 2010)
c.2872C>A	p.R958S	missense	1	Paranoid SCZ	F	n.a.	(Peykov et al., 2015)
c.3060G>A	p.E1020E	synonymous	1	ASD	n.a.	n.a.	(Berkel et al., 2010)
c.3189G>A	p.P1063P	synonymous	1	ASD	n.a.	n.a.	(Berkel et al., 2010)

Supplementary Table 1b

Mutation	Affected amino acid	Variant type	Number of patients	Phenotype	Sex	Transmission source	Reference
c.3355C>A	p.P1119T	missense	1	Schizoaffective	F	n.a.	(Peykov et al., 2015)
c.3431C>T	p.P1144L	missense	1	Paranoid SCZ	M	Mother	(Peykov et al., 2015)
c.3484G>A	p.E1162K	missense	1	Pervasive developmental disorder not otherwise specified	n.a.	n.a.	(Leblond et al., 2012)
c.3508G>A	p.G1170R	missense	1	Autism with ID and epilepsy	M	Mother	(Leblond et al., 2012)
c.3846C>T	p.Y1282Y	synonymous	1	SCZ	n.a.	n.a.	(Peykov et al., 2015)
c.3960C>T	p.D1320D	synonymous	1	ASD	n.a.	n.a.	(Berkel et al., 2010)
c.4113C>T	p.P1371P	synonymous	1	ASD	n.a.	n.a.	Berkel et al., 2010
c.4126G>A	p.V1376I	missense	1	Autism	M	Mother	(Leblond et al., 2012)
c.4161-66dup	p.L1387 P1388dup	inframe-insertion	1	ASD	M	Mother	(Berkel et al., 2010)
c.4279C>T	p.R1427W	missense	1	ID	F	Mother	(Berkel et al., 2010)
c.4287T>C	P.A1429A	synonymous	1	SCZ	n.a.	n.a.	(Peykov et al., 2015)
c.4306GinsCA	p.D1436A	frameshift	1	Autism	M	*de novo*	(Sanders et al., 2012)
c.4517C>T	p.T1506M	missense	1	Autism	M	Mother	(Berkel et al., 2010)
c.4603G>A	p.D1535N	missense	1	Autism with ID	M	Mother	(Leblond et al., 2012)
c.4822G>A	p.V1608I	missense	1	Residual SCZ	M	Mother	(Peykov et al., 2015)
c.4926G>A	p.P1642P	synonymous	1	SCZ	n.a.	n.a.	(Peykov et al., 2015)
c.4936C>A	p.L1646M	missense	1	Paranoid SCZ	M	Mother	(Peykov et al., 2015)
c.5165T>C	p.L1722P	missense	1	Autism with ID	M	Father	(Leblond et al., 2012)
c.5185G>A	p.A1729T	missense	1	Autism	M	Mother	(Berkel et al., 2010)
c.5191G>A	p.A1731T	missense	1	ASD	M	*de novo*	(Yuen et al., 2017)
c.5191G>T	p.A1731S	missense	4	3 Paranoid SCZ, 1 Disorganized SCZ	2M, 2F	Mother	(Peykov et al., 2015)
c.5289G>A	p.S1763S	synonymous	1	ID	n.a.	n.a.	(Berkel et al., 2010)

Supplementary Table 1a and b: Summary of *SHANK2* gene variants in the coding sequence identified in patients with neurodevelopmental and neuropsychiatric disorders but not in healthy human controls. (NCBI reference sequence: NM_012309.4). Data from Table 2 are included and highlighted in grey. n.a., not available. ID = Intellectual disability; ASD = Autism spectrum disorder; SCZ = Schizophrenia.

Supplementary Table 2a

	Δex15-16	*Δex15-16 Pcp2-Cre*	*Δex15-16 CaMK2a-Cre*	*Δex15-16 Viaat-Cre*	*Δex16*	*Δex16 L7–Cre*	*Δex24*	*Δex24 Pcp2-Cre*	*Δex24 Emx1-Cre*	*Δex24 CaMK2a-Cre*	*rAAV R462X*
**Genetic**	Global exon 15-16 deletion leading to a frameshift and PDZ removal	Exon 15-16 deletion specifically in PC	Exon 15-16 deletion specifically in excitatory neurons	Exon 15-16 deletion specifically in GABAergic inhibitory neurons	Global exon 16 deletion leading to a frameshift and PDZ removal	Exon 16 deletion specifically in Purkinje cells (PC)	Global exon 24 deletion causing proline-rich region deletion	Exon 24 deletion specifically in Purkinje cells (PC)	Exon 24 deletion specifically in neocortex and hippocampus (Hippo.)	Exon 24 deletion specifically in the forebrain	High and low overexpression of human SHANK2-A^{R462X} in forebrain
**Genetic background**	129/SvJ ES backcrossed to C57BL/6N for > 5 generations	Crossing Pcp2-Cre with $Shank2^{fl/+}$ (C57BL/6J) and then crossing with *Shank2*$^{fl/fl}$	Crossing CAMK2a-Cre with $Shank2^{fl/fl}$ (C57BL/6J)	Crossing Viaat-Cre with $Shank2^{fl/fl}$ (C57BL/6J)	129 R1-ES backcrossed to C57BL/6J for 10-11 generations	Crossing L7(Pcp2)-Cre with *Shank2*$^{fl/fl}$	129 R1-ES backcrossed to C57BL/6J mice for > 5 generations	Crossing Pcp2-Cre with *Shank2*$^{\Delta ex24fl/\Delta ex24fl}$	Crossing Emx1-Cre with *Shank2*$^{\Delta ex24fl/\Delta ex24fl}$	Crossing CaMk2a-cre with *Shank2*$^{\Delta ex24fl/\Delta ex24fl}$	C57BL6/N mice (Charles River)
**Expressed isoforms**	Predicted to be none	Predicted to be none only in PC	Predicted to be none only in excitatory neurons	Predicted to be none only in GABAergic inhibitory neurons	Predicted to be none	Predicted to be none only in PC	Not known	Not known	Not known	Not known	Predicted to be all expressed
**Age of mice**	Biochem. 8-12W<br>Morphol. 8-9W<br>Ephys. 3-9W<br>Behavior 1-5M<br><br>Biochem. cerebellum ≈ P20<br>Ephys. cerebellum ≈ P20<br>Behavior cerebellum 5-6M	Biochem. ≈ P20<br>Ephys. ≈ P21<br>Behavior 2-6M	Biochem. 3-6M<br>Ephys. P22-26<br>Behavior 9-17W	Biochem. 2-4M<br>Ephys. P21-34<br>Behavior 9-15W	Biochem. P25 & P70<br>Morphol. E18 neuron culture<br>Morphol. adult mice<br>Ephys. P21-28<br>Behavior 6-8M<br><br>Biochem. cerebellum adult<br>Ephys. cerebellum P9-P35	Ephys. P9-P35<br>Behavior 8-16W	Biochem 2-4M<br>Ephys. 2-4M<br>Behavior adult	Behavior adult	Behavior adult	Behavior adult	Ephys. ≈ P69 & P90<br>Behavior 3-4M
**Overall appearance**	No change in body weight and normal neuronal cell number in the brain	No change in body weight	No change in body weight	No change in body weight	Body weight was reduced but normal appearance and overall brain morphology	n.a.	No apparent developmental defects and no difference in body weight; No spontaneous seizures	n.a.	n.a.	n.a.	No change in body weight
**E-Phys. analysis**	**Hippo.CA1**<br>Normal<br>- synaptic transmission<br>- mEPSC freq.<br>-mEPSC amp.<br>- mGluR-LTD<br>Reduced<br>- NMDA/AMPA<br>- NMDAR-LTP<br>- NMDAR-LTD<br>**Cerebel.**:<br>**PF-PC**:<br>Normal<br>- mEPSC amp.<br>- LTD<br>Reduced<br>- mEPSC freq.	**Cerebellum**:<br>**PF-PC**:<br>Normal<br>- mEPSC amp.<br>- LTD<br>Reduced<br>- mEPSC freq.	**Hippo.CA1**<br>Normal<br>- mEPSCs amp.<br>- mIPSCs amp.<br>- mIPSCs freq.<br>- paired pulse facilitation<br><br>Reduced<br>- mEPSCs freq.	**Hippo.CA1**<br>Normal<br>- mEPSCs amp.<br>- mEPSCs freq.<br>- mIPSCs amp.<br>- mIPSCs freq.<br>**Striatum**<br>Normal<br>- mEPSC amp.<br>- mEPSC freq.<br><br>Reduced<br>- mIPSC amp.<br>- mIPSC freq.<br>**Striatum without tetrodotoxin**<br>Normal<br>- sIPSC freq.<br>- sEPSC amp.<br><br>Reduced<br>- sIPSC amp.<br>-sEPSC freq.	**Hippo.CA1**<br>Normal<br>- mEPSC amp.<br>- mIPSC freq.<br>- sEPSC<br>- LTD<br>Reduced<br>- synaptic transmission<br>- mEPSC freq.<br>- I/O ratio<br>- mIPSC amp.<br>Increased<br>-NMDA/AMPA<br>-LTP<br>**Cerbel.**(*PC*)<br>Normal<br>- excitability<br>- EPSCs<br>- sIPSCs amp.<br>Increased<br>- sIPSCs freq.<br>- spike Irregularity and impaired plasticity	**Cerebellum**:<br>***anterior lobules***<br>-Irregular simple spiking<br>-Increased coefficient of variation2 (CV2)<br>***posterior lobules & flocculus***<br>-Irregular simple spiking increased in both CV1 and CV2<br>-Normal simple spike firing freq. and complex spike duration and freq.	**Hippo.**PSDs<br>Reduced<br>- NMDA/AMPA<br>- NMDA currents<br>- NMDAR mediated eEPSC<br>Increased<br>- AMPAR mediated EPSC	n.a.	n.a.	n.a.	**Hippo.CA1**<br>Normal<br>- mIPSC amp.<br>- mIPSC freq.<br>Reduced<br>- mEPSC amp.<br>**Entorhinal cortex layer 2/3**<br>Normal<br>- mIPSC amp.<br>- mIPSC freq.<br>- mEPSC freq.<br>Reduced<br>- mEPSC amp.
**References**	(Won et al., 2012; Ha et al., 2016; Lim et al., 2017; Kim et al., 2018)	(Ha et al., 2016)	(Kim et al., 2018)	(Kim et al., 2018)	(Schmeisser et al., 2012; Peter et al., 2016; Lim et al., 2017)	(Peter et al., 2016)	(Pappas et al., 2017)	(Pappas et al., 2017)	(Pappas et al., 2017)	(Pappas et al., 2017)	(Berkel et al., 2012)

Supplementary Table 2b

	Δex15-16	*Δex15-16 Pcp2-Cre*	*Δex15-16 CaMK2a-Cre*	*Δex15-16 Viaat-Cre*	*Δex16*	*Δex16 L7–Cre*	*Δex24*	*Δex24 Pcp2-Cre*	*Δex24 Emx1-Cre*	*Δex24 CaMK2a-Cre*	*rAAV R462X*
**Spines & Synapses (Structure)**	**Hippo.**<br>- Normal spines density, number and length<br>**Cerebellum**<br>- Reduced PSD numbers<br>- Increased mis-matched excitatory synapses and free dendritic spines	n.a.	n.a.	n.a.	**Hippo.**<br>- Reduced spine density and number<br>**Cerebellum**<br>- Normal PSD thickness and length<br>Normal spine density, length and width	n.a	**Hippo.**<br>- Reduced dark part of PSD at CA1 synapses<br>**Cerebellum & striatum**<br>- Normal PSD structure	n.a	n.a.	n.a.	- Densely packed filopodia structures instead of mature spines<br>- Increased size and number of AMPAR clusters
**Synaptic proteins**	**Whole brain**<br>- Reduced<br>p-CaMKIIα/β<br>p-ERK1/2<br>p38, p-GluA1<br>- Increased<br>GluN1<br>- Normal<br>p-PAK1/3<br>p-mTOR<br>GluN2A<br>GluA2<br>PSD-95<br>mGluR1/5<br>SAP97<br>GKAP,<br>SynGAP1<br>Homer1<br>GKAP<br>GIT1<br>PLC-b3<br>Shank1/3<br>**Hippo.**<br>-Reduced<br>GABAA-R a2<br>GluN1<br>**Cerebellum**<br>- Reduced<br>***lysate & synapt.***<br>GluA2/GluD2<br>VGluT1<br>PSD93,Homer<br>***lysates***<br>GluN1,GluA2/3<br>mGluR1,IP3R<br>Nlgn1<br>CaMKIIα/β<br>***synaptosomes***<br>GluA1,GluN2C<br>Gephyrin<br>PSD95<br>-Normal<br>VGAT & GAD65	**Cerebellum**<br>- Reduced<br>***synaptosomes***<br>GluD2<br>PSD93<br>***lysates***<br>GluD2<br>GluA1<br>Homer	n.a.	n.a.	**Hippo.**<br>- Increased<br>GluN1<br>GluN2B<br>GluA1<br>PSD95<br>**Striatum:**<br>- Increased<br>GluN1<br>GluN2A<br>GluA2<br>Shank3<br>**Cerebellum**<br>***synaptosomes***<br>- Reduced<br>GluA1<br>GluA2<br>- Normal<br>Nlgn3	n.a.	**Hippo.** PSD<br>- Reduced<br>GluN1<br>- Increased<br>GluN2A<br>GluN2B<br>- Normal<br>GluN2C<br>GluN2D<br>GluA1<br>GluA2<br>CaMKII	n.a.	n.a.	n.a.	**Hippo.**<br>- Normal<br>GluA1<br>GluA2<br>pGluA1-S831<br>pGluA1-S845
**General behavior and motor function**	- Hyper-activity in the open field and LABORAS<br>- Normal olfaction Impaired nesting Decreased digging<br>- Impaired pup retrieval<br>- Suppressed motor coordination in Erasmus ladder	- Impaired motor coordination in Erasmus ladder<br>- Normal motor performance in the rotarod test	- Mild hyper-activity in open field<br>- Hyper-activity in LABORAS in the first 12 h but not later	- Hyper-activity in open field<br>- Hyper-activity in LABORAS in the first 6 h but not later	- Hyper-activity in the open field<br>- Normal olfaction<br>- Normal motor coordination<br>- Short digging bouts	- No hyper-activity<br>- Normal baseline of motor performance in Erasmus ladder	- Hyper-activity in the home cage and open field<br>- Impaired motor performance in the rotarod test	- No hyper-activity in the open field<br>- Impaired motor performance in the rotarod test	- Hyper-activity in the open field<br>- Normal motor performance in the rotarod test	- No hyper-activity in the open field	- Regular locomotor behavior in the open field and the rotarod tests
**References**	(Won et al., 2012; Ha et al., 2016; Lim et al., 2017; Kim et al., 2018)	(Ha et al., 2016)	(Kim et al., 2018)	(Kim et al., 2018)	(Schmeisser et al., 2012; Peter et al., 2016; Lim et al., 2017)	(Peter et al., 2016)	(Pappas et al., 2017)	(Pappas et al., 2017)	(Pappas et al., 2017)	(Pappas et al., 2017)	(Berkel et al., 2012)

Supplementary Table 2c

	Δex15-16	*Δex15-16 Pcp2-Cre*	*Δex15-16 CaMK2a-Cre*	*Δex15-16 Viaat-Cre*	*Δex16*	*Δex16 L7–Cre*	*Δex24*	*Δex24 Pcp2-Cre*	*Δex24 Emx1-Cre*	*Δex24 CaMK2a-Cre*	*rAAV R462X*
**Anxiety**	- Increased anxiety in elevated plus maze<br>- Normal behavior in the center region of an open-field arena<br>- Normal behavior in the light-dark test.	- Mildly increased anxiety in the light-dark test<br>- Normal behavior in elevated plus maze<br>- Normal freq. in visiting the center quadrant in the open-field	- Increased anxiety behavior in the open field and light-dark box<br>- Normal behavior in elevated plus maze	- Normal behavior in open field, elevated plus maze and light-dark box	- Increase anxiety in the light-dark test	- Normal behavior in the open field	n.a.	n.a.	n.a.	n.a.	- Normal behavior in the open field and light dark box
**Repetitive behavior**	- Increase jumping and upright scrabbling<br>- Normal grooming in the home cage and LABORAS<br>- Increase grooming in novel object recognition arena<br>- Normal hole-board repetitive behavior	- Normal grooming, jumping, digging and marble burying<br>- Increased repetitive behavior in the hole-board	- Normal grooming in home cage and LABORAS test<br>- Normal behavior in hole board test<br>- Normal jumping counts<br>- Reduced digging	- Increased grooming in home cage and LABORAS test<br>- Increased repetitive behavior in hole board test<br>- Normal jumping counts<br>- Normal digging	- Increased grooming in female and the stereotype behavior was not severe	- Normal in marble burying or in the duration of grooming<br>- Increased repetitive behavior in the T-maze	- Decrease in the duration and the number of bouts of grooming<br>- Increased visits in the hole-board but no differences in the total number of head pokes	n.a.	n.a.	n.a.	n.a.
**Learning, memory and cognition**	- Partially impaired spatial learning and memory in the Morris water maze<br>- Normal novel object recognition	- Impaired motor learning in Erasmus ladder test	n.a.	n.a.	- Normal working memory or novel object recognition<br>- Mild deficit of spatial memory deficit in the Morris water maze	- Impaired motor learning in Erasmus ladder test, compensatoy eye movements and Pavlovian eye-blink	- Impaired spatial learning and memory in the Morris water maze<br>- Impaired cognitive function	n.a.	- Impaired spatial learning and memory in the Morris water maze	n.a.	- Impaired cognition in the puzzle box<br>- Impaired Novel object recognition<br>- Difference in high and low level SHANK2A^{R462X} mice
**Social interaction**	*Home cage interaction*<br>Reduced interaction with normal target<br><br>*Three chamber social test*<br>Less preference interaction towards a mouse over an inanimate object but Normal social novelty recognition	*Direct social interaction*<br>Normal social interaction and normal social novelty recognition<br><br>*Three chamber social test*<br>Normal social interaction and normal social novelty recognition	*Three chamber social test*<br>Reduced social interaction and reduced preference index<br>But Normal novelty social recognition<br><br>*Direct social interaction*<br>Reduced levels of male-female social interaction	*Three chamber social test*<br>Normal social interaction and novelty social recognition<br><br>*Direct social interaction*<br>Reduced levels of male-female social interaction	*Free same sex interaction*<br>No difference in latency for the first contact but difficulty in maintaining social contacts.<br><br>*Free opposite sex interaction*<br>Longer latency for the first contact but no impairment in contact maintaining<br><br>*Three chamber social test*<br>Reduced conspecific recognition or interest for social novelty	*Three chamber social test*<br>Deficits in social interaction and impaired social novelty recognition	*Social affiliateion test*<br>Normal social preference score in males and females<br><br>*Social dyadic test*<br>No distinctions between bi-directional and uni-directional interactions	n.a.	*Social affiliateion test*<br>Significant high social preference score in females but borderline higher in males	n.a.	n.a.
**References**	(Won et al., 2012; Ha et al., 2016; Lim et al., 2017; Kim et al., 2018)	(Ha et al., 2016)	(Kim et al., 2018)	(Kim et al., 2018)	(Schmeisser et al., 2012; Peter et al., 2016; Lim et al., 2017)	(Peter et al., 2016)	(Pappas et al., 2017)	(Pappas et al., 2017)	(Pappas et al., 2017)	(Pappas et al., 2017)	(Berkel et al., 2012)

Supplementary Table 2d

	Δex15-16	Δex15-16 Pcp2-Cre	Δex15-16 CaMK2a-Cre	Δex15-16 Viaat-Cre	Δex16	Δex16 L7–Cre	Δex24	Δex24 Pcp2-Cre	Δex24 Emx1-Cre	Δex24 CaMK2a-Cre	rAAV R462X
**Mania like behavior, depression & schizo-phrenia**	n.a.	n.a.	n.a.	n.a.	n.a.	n.a.	- Normal forced swim tests; tail suspension<br>- Enhanced reward-seeking<br>- Disturbed circadian rhythms<br>- Bipolar and mania like Anhedonia-in the sucrose preference<br>- Normal Schizophrenic like behavior in prepulse inhibition	n.a.	- Mania like behavior	n.a.	n.a.
**Ultrasonic vocalization (USV)**	- Less courtship USVs<br>- Longer latency for the first call in male mice	- Normal number of USVs<br>- Reduced USVs freq. induced by adult male-female interaction<br>- Normal Numbers of USVs in pups separated from their mothers	- Normal number of USVs but increased latency to first call during courtship	- Reduced numbers of USVs and increased the latency first call	- Normal number of USVs during male–male contact<br>- Increased first USV latency In the socio-sexual context of a males; unstructured calls<br>- Increased latency for the first call and reduced USV during female–female contact; short and un-structured calls<br>- Increased USVs in female pups at P4 & P10	n.a.	n.a.	n.a.	n.a.	n.a.	n.a.
**References**	(Won et al., 2012; Ha et al., 2016; Lim et al., 2017; Kim et al., 2018)	(Ha et al., 2016)	(Kim et al., 2018)	(Kim et al., 2018)	(Schmeisser et al., 2012; Peter et al., 2016; Lim et al., 2017)	(Peter et al., 2016)	(Pappas et al., 2017)	(Pappas et al., 2017)	(Pappas et al., 2017)	(Pappas et al., 2017)	(Berkel et al., 2012)

Supplementary Table 2a–d: Phenotypes of different global and conditional *Shank2* knockout mice and of mice with rAAV-mediated overexpression of truncated SHANK2A isoform. Hippo. = Hippocampus; PC = Purkinje cells; PF = parallel fibers; amp. = amplitude; freq. = frequency; USV = ultrasonic vocalization.

References

Berkel, S., Marshall, C.R., Weiss, B., Howe, J., Roeth, R., Moog, U., et al. (2010). Mutations in the SHANK2 synaptic scaffolding gene in autism spectrum disorder and mental retardation. *Nat Genet* 42**,** 489-491. doi: 10.1038/ng.589.

Berkel, S., Tang, W., Trevino, M., Vogt, M., Obenhaus, H.A., Gass, P., et al. (2012). Inherited and de novo SHANK2 variants associated with autism spectrum disorder impair neuronal morphogenesis and physiology. *Hum Mol Genet* 21**,** 344-357. doi: 10.1093/hmg/ddr470.

Bowling, K.M., Thompson, M.L., Amaral, M.D., Finnila, C.R., Hiatt, S.M., Engel, K.L., et al. (2017). Genomic diagnosis for children with intellectual disability and/or developmental delay. *Genome Med* 9**,** 43. doi: 10.1186/s13073-017-0433-1.

Ha, S., Lee, D., Cho, Y.S., Chung, C., Yoo, Y.E., Kim, J., et al. (2016). Cerebellar Shank2 Regulates Excitatory Synapse Density, Motor Coordination, and Specific Repetitive and Anxiety-Like Behaviors. *J Neurosci* 36**,** 12129-12143. doi: 10.1523/JNEUROSCI.1849-16.2016.

Homann, O.R., Misura, K., Lamas, E., Sandrock, R.W., Nelson, P., McDonough, S.I., et al. (2016). Whole-genome sequencing in multiplex families with psychoses reveals mutations in the SHANK2 and SMARCA1 genes segregating with illness. *Mol Psychiatry* 21**,** 1690-1695. doi: 10.1038/mp.2016.24.

Kim, R., Kim, J., Chung, C., Ha, S., Lee, S., Lee, E., et al. (2018). Cell-Type-Specific Shank2 Deletion in Mice Leads to Differential Synaptic and Behavioral Phenotypes. *J Neurosci* 38**,** 4076-4092. doi: 10.1523/JNEUROSCI.2684-17.2018.

Leblond, C.S., Heinrich, J., Delorme, R., Proepper, C., Betancur, C., Huguet, G., et al. (2012). Genetic and functional analyses of SHANK2 mutations suggest a multiple hit model of autism spectrum disorders. *PLoS Genet* 8**,** e1002521. doi: 10.1371/journal.pgen.1002521.

Lim, C.S., Kim, H., Yu, N.K., Kang, S.J., Kim, T., Ko, H.G., et al. (2017). Enhancing inhibitory synaptic function reverses spatial memory deficits in Shank2 mutant mice. *Neuropharmacology* 112**,** 104-112. doi: 10.1016/j.neuropharm.2016.08.016.

Pappas, A.L., Bey, A.L., Wang, X., Rossi, M., Kim, Y.H., Yan, H., et al. (2017). Deficiency of Shank2 causes mania-like behavior that responds to mood stabilizers. *JCI Insight* 2. doi: 10.1172/jci.insight.92052.

Peter, S., Ten Brinke, M.M., Stedehouder, J., Reinelt, C.M., Wu, B., Zhou, H., et al. (2016). Dysfunctional cerebellar Purkinje cells contribute to autism-like behaviour in Shank2-deficient mice. *Nat Commun* 7**,** 12627. doi: 10.1038/ncomms12627.

Peykov, S., Berkel, S., Schoen, M., Weiss, K., Degenhardt, F., Strohmaier, J., et al. (2015). Identification and functional characterization of rare SHANK2 variants in schizophrenia. *Mol Psychiatry* 20**,** 1489-1498. doi: 10.1038/mp.2014.172.

Rauch, A., Wieczorek, D., Graf, E., Wieland, T., Endele, S., Schwarzmayr, T., et al. (2012). Range of genetic mutations associated with severe non-syndromic sporadic intellectual disability: an exome sequencing study. *Lancet* 380**,** 1674-1682. doi: 10.1016/S0140-6736(12)61480-9.

Sanders, S.J., Murtha, M.T., Gupta, A.R., Murdoch, J.D., Raubeson, M.J., Willsey, A.J., et al. (2012). De novo mutations revealed by whole-exome sequencing are strongly associated with autism. *Nature* 485**,** 237-241. doi: 10.1038/nature10945.

Schmeisser, M.J., Ey, E., Wegener, S., Bockmann, J., Stempel, A.V., Kuebler, A., et al. (2012). Autistic-like behaviours and hyperactivity in mice lacking ProSAP1/Shank2. *Nature* 486**,** 256-260. 1015.doi: 10.1038/nature1

Won, H., Lee, H.R., Gee, H.Y., Mah, W., Kim, J.I., Lee, J., et al. (2012). Autistic-like social behaviour in Shank2-mutant mice improved by restoring NMDA receptor function. *Nature* 486**,** 261-265. doi: 10.1038/nature11208.

Yuen, R.KC., Merico, D., Bookman, M., J, L.H., Thiruvahindrapuram, B., Patel, R.V., et al. (2017). Whole genome sequencing resource identifies 18 new candidate genes for autism spectrum disorder. *Nat Neurosci* 20**,** 602-611. doi: 10.1038/nn.4524.

Yuen, R.KC., Merico, D., Cao, H., Pellecchia, G., Alipanahi, B., Thiruvahindrapuram, B., et al. (2016). Genome-wide characteristics of de novo mutations in autism. *NPJ Genom Med* 1**,** 160271-1602710. doi: 10.1038/npjgenmed.2016.27.
